# Dietary Patterns and Health Outcomes among African American Maintenance Hemodialysis Patients

**DOI:** 10.3390/nu12030797

**Published:** 2020-03-18

**Authors:** Dina A. Tallman, Eno Latifi, Deepinder Kaur, Ayesha Sulaheen, T. Alp Ikizler, Karuthan Chinna, Zulfitri Azuan Mat Daud, Tilakavati Karupaiah, Pramod Khosla

**Affiliations:** 1 Department of Nutrition and Food Science, Wayne State University, Detroit, MI 48202, USAEno.Latifi@wayne.edu (E.L.); kdeepinder@wayne.edu (D.K.); 2 Dietetics Program, Faculty of Health Sciences, University Kebangsaan Malaysia, 50300 Kuala Lumpur, Malaysia; aishaltaf@ymail.com; 3 Division of Nephrology and Hypertension, Vanderbilt University Medical Center, Nashville, TN 37232, USA; alp.ikizler@vanderbilt.edu; 4 School of Medicine, Faculty of Health Sciences, Taylors University, 47500 Subang Jaya, Malaysia; karuthan@gmail.com (K.C.); tilly_karu@yahoo.co.uk (T.K.); 5 Department of Nutrition and Dietetics, Faculty of Medicine and Health Sciences, University Putra Malaysia, 43400 Serdang, Malaysia; zulfitri@upm.edu.my

**Keywords:** hemodialysis, maintenance hemodialysis, dietary patterns, cluster analysis, quality of life, lipoproteins, inflammation

## Abstract

The association between dietary patterns and health outcomes, such as quality of life (QOL), in maintenance hemodialysis (MHD) patients with certain racial backgrounds has not been studied in detail. QOL is a powerful outcome measure in which dietary patterns could be a modifying factor. This study is a secondary analysis examining the association between dietary patterns and health outcomes in 101 African American (AA) maintenance hemodialysis (MHD) patients participating in the Palm Tocotrienols in Chronic Hemodialysis (PATCH) study. Quality of life (QOL) was assessed using the Kidney Disease Quality of Life 36-item survey (KDQOL-36™). Blood samples were analyzed for lipids, lipoprotein subfractions, and inflammatory markers. Food intake was measured using six non-consecutive 24-h dietary recalls over 15 months. Implausible energy intake reports were screened out by comparing reported energy intake (rEI) with predicted total energy expenditure (pTEE). Cluster analysis, using the *k*-means algorithm, identified two distinct dietary patterns in the study population: a high “sugar sweetened beverage” pattern (hiSSB) and a low “sugar sweetened beverage pattern” (loSSB). In the hiSSB group, consumption of SSB accounted for ~28% of energy intake, while SSB represented only 9% of energy intake in the loSSB group. The hiSSB group was characterized by a higher intake of total calories, sugar and percentage of kilocalories from carbohydrates, whereas the percentage of kilocalories from protein and fat was lower. While additional micronutrient intakes differed between groups (vitamin C, zinc, chromium), these were significantly lower than recommended values in the entire cohort. Patients in the hiSSB group presented with lower high-density lipoprotein cholesterol (HDL-C), lower large HDL particles and smaller low density lipoprotein (LDL) particle diameters. Antidepressant usage was significantly higher in the hiSSB group. Patients in the hiSSB group scored lower across all five KDQOL domains and scored significantly lower in the mental composite domain. MHD patients following a hiSSB dietary pattern had smaller dense LDL particles, lower HDL-C, and a lower QOL. Suboptimal intakes of fruits, vegetables, and grains as well as key micronutrients were evident in both patterns.

## 1. Introduction

The global prevalence of chronic kidney disease (CKD) is estimated to be 11%–13%, [1] affecting approximately 30 million Americans. With an increasing prevalence of CKD, the impact of treatment is projected to confer a significant economic and social burden [2]. In the United States, African Americans (AA) suffer a disproportionate burden of end stage renal disease (ESRD), comprising 35% of all dialysis patients, as they are 3.7 times more likely to progress to ESRD than whites [3,4]. This increase in risk is partially attributed to higher rates of hypertension (HTN), diabetes mellitus (DM), and cardiovascular disease [5]. Primary prevention strategies focused on preventing the development of CKD include dietary modification. “Unhealthy” dietary patterns, such as those high in fat and sugar [6], have been associated with significantly increased CKD incidence and progression risk [7], whereas healthier diet patterns, which include higher intakes of vegetables, fruits, legumes, nuts, whole grains, fish, and low-fat dairy, and lower intakes of red and processed meats, sodium, and sugar-sweetened beverages, have been associated with a lower incidence of CKD [8].

Patients requiring maintenance hemodialysis (MHD) are expected to make major lifestyle changes, including adherence to a conventional renal diet limited in fruits, vegetables, nuts, legumes, dairy, and whole grains due to concerns about both phosphorus and potassium [9]. However, there are few studies examining the association between dietary patterns and health outcomes, especially for the AA ESRD population [10].

Two common analytical approaches used to identify dietary patterns in nutritional epidemiology are à priori and à posteriori methods [11]. À priori methods are based on indices of diet quality or scores defined by nutritional health, whereas à posteriori methods, such as factor and cluster analyses, use multivariate statistical techniques to derive dietary patterns [12]. By reducing diet data into mutually exclusive patterns, a cluster analysis approach separates individuals with similar mean dietary intakes into non-overlapping groups from which health outcomes can be compared [13,14]. This study therefore applied an à posteriori approach to evaluate dietary patterns of AA patients on MHD (AA-MHD) to understand the impact of diet on health outcomes. 

## 2. Materials and Methods

### 2.1. Subjects

Data were collected by trained research personnel from subjects participating in the Palm Tocotrienols in Chronic Hemodialysis (PATCH) Study (NCT02358967) and were extracted for the secondary analysis of the current study. Briefly, the PATCH Study was a randomized, double-blind, placebo-controlled trial evaluating the effects of daily supplementation with 300 mg of a vitamin E tocotrienol-rich fraction (TRF) on markers of inflammation, oxidative stress, and blood lipids in patients undergoing MHD at multiple dialysis clinics, both within Michigan, USA, and overseas. The criteria for study participation included patients with ESRD receiving thrice-weekly hemodialysis for at least 120 days, aged over 18 years, with a life expectancy of over one year, and the ability to understand and provide informed consent. Exclusion criteria included a history of poor adherence to hemodialysis, active malignancy, AIDS, and patients receiving nutritional support. This current report is based on the data collected from the Michigan cohort (135 subjects enrolled). Subjects were enrolled between June 2017 and February 2018, with all study procedures completed by April 2019. The study was approved by the ethics boards of participating dialysis units and Wayne State University’s Institutional Review Board. All subjects provided written informed consent.

### 2.2. Collection of Clinical Information

We used baseline clinical laboratory data, heights, and post-dialysis weights, which were obtained from patients’ medical records collected from the original study. Body mass index was calculated based on Quetelet’s Index [15].

### 2.3. Blood Sampling and Lipid Measurement

In the original study, pre-dialysis non-fasting blood samples were collected in Ethylenediaminetetraacetic acid (EDTA) or lithium heparin tubes and kept on ice. Plasma samples were transported to Wayne State University within two hours and centrifuged at 3500 rpm for 10 min to separate plasma. Aliquots were stored at −80 °C. C-Reactive Protein (CRP), Interleukin 6 (IL-6), Interleukin 18 (IL-18), and Monocyte Chemoattractant Protein-1 (MCP-1) samples were analyzed in duplicates in 384-well AlphaPlates^TM^ (PerkinElmer®). IL-6 values lower than the detection limit (1.3 pg/mL) were assigned a value of 0.01 pg/mL. The plasma total cholesterol (TC) and triglycerides (TAG) were determined by enzymatic assays (Pointe Scientific Inc., Canton, MI, USA). High density lipoprotein cholesterol (HDL-C) was measured in the supernatant after precipitation of apoB-containing lipoproteins by dextran sulfate and magnesium ions (Pointe Scientific Inc., Canton, MI, USA). Low density lipoprotein cholesterol (LDLC) was calculated using the Friedwald equation by difference (LDLC = TC − HDLC − TAG/5). HDL and LDL subfractions from plasma were also measured via polyacrylamide gel electrophoresis using the Lipoprint^TM^ System (Quantimetrix Corporation, Redondo Beach, CA, USA). Using the manufacturer’s proprietary software, HDL and LDL subfractions were quantitated after electrophoresis. Both HDL and LDL were then grouped into large, intermediate, and small subfractions. The Lipoprint^TM^ system is U.S. Food and Drug Administration (FDA) certified for LDL measurements; however, values for HDL are for research purposes only.

### 2.4. Assessment of Kidney Disease Quality of Life (KDQOL)

As a measure of health-related quality of life (HRQOL), Kidney Disease Quality of Life 36-item surveys (KDQOL-36™) were administered at baseline [16,17] by the same research team member for a subset of the original study population. The KDQOL-36 is comprised of five subscales calculated separately: 1) SF-12 physical component summary (PCS), 2) SF-12 mental component summary (MCS), 3) burden of kidney disease, 4) symptoms of kidney disease, and 5) effects of kidney disease. Subscale scores range from 0 to 100, with lower scores indicating poor self-reported QOL [18,19].

### 2.5. Assessment of Dietary Intake

In the original study, six 24-h dietary recalls taken on non-dialysis days were collected in person by the same registered dietitian quarterly over a 15-month time period [20,21] using the U.S. Department of Agriculture (USDA) five-pass method [22]. Reported energy intake (rEI) and nutrient analysis were calculated using the Food Processor SQL software package (version 11.2, 2016, ESHA Research, Salem, OR, USA). To reduce the effect of confounding from physiologically implausible rEI, dietary reports from over and under-reporters were screened out [23]. Using the method introduced by McCrory et al., which accounts for within-subject errors in rEI and predicted total energy expenditure (pTEE) without estimation of physical activity level, a 2 standard deviation cutoff was used to classify 24HR less than 56% or more than 144% of estimated energy needs as implausible [24]. Edema-free adjusted body weight was used for patients whose weight was less than 95% or greater than 115% of standard body weight [25].

### 2.6. Statistical Analysis

Diet analyses revealed thirty-three food groups, which were converted to percent contribution of total daily energy (%TE) intakes [26]. Cluster analysis was performed using the *k*-means algorithm, a nonhierarchical clustering method which classifies participants into non-overlapping groups based on Euclidean distance. A set of two clusters was selected as the solution that provided a sample size large enough to allow for analyses of distinct groups. Cluster membership was concordant for 84.2% of all subjects in clusters 1 and 2 (κ = 0.674). The two clusters were named according to the food group providing the greatest total energy intake (hiSSB and loSSB) [12,27].

Differences in nutrient intakes, health characteristics, and KDQOL across the food intake patterns were compared according to data distribution, using the t test or Mann–Whitney U test for continuous variables and Pearson chi-square test for categorical variables. For all tests, the level of significance was set as *p* < 0.05. All analyses were performed using SPSS (version 25, SPSS Inc., Chicago, IL, USA).

## 3. Results

### 3.1. Clusters Identified for Dietary Patterns

We identified two major dietary patterns: cluster 1, with a high “sugar sweetened beverage” pattern (hiSSB) and a low “sugar sweetened beverage” pattern (loSSB). As illustrated in Table 1, the hiSSB dietary pattern was characterized by higher energy contributions from calorically sweetened soft and juice drinks (*p* < 0.001) and poultry (*p* < 0.05), whereas the greatest energy contributors to the loSSB group were unprocessed red meat (*p* < 0.05), fish and shellfish, (*p* < 0.05), and custard style desserts such as puddings, ice cream, and cheesecake (*p* < 0.05). A total of 47 patients were classified in the hiSSB pattern and 54 in the loSSB pattern.

### 3.2. Baseline Characteristics

Subjects were 100% African American, 59% male, aged 25–87, and with an average Body Mass Index (BMI) of 27 kg/m^2^. About 2/3 had a diagnosis of DM and 1/3 were tobacco users (Table 2).

### 3.3. Characteristics of Patients According to Dietary Cluster

We compared the baseline characteristics of the subjects by diet clusters. To minimize statistical bias as an influential outlier, one subject with hypertriglyceridemia (plasma TG 943 mg/dL) was excluded in subsequent analyses. Kidney specific clinical parameters did not differ significantly between the two diet clusters. Patients in the hiSSB group had a significantly higher BMI and were more likely to be prescribed an antidepressant than those patients in the loSSB group. Both total HDL cholesterol and large HDL subfractions were significantly lower in the hiSSB group than in the loSSB group. Patients in the hiSSB group were 2.4 times more likely to have a pattern B phenotype, characterized by predominantly smaller and denser LDL subfractions. The hiSSB dietary pattern comparatively, although not significantly, tended towards higher inflammatory markers for CRP, IL-18, and MCP-1 (Table 2).

### 3.4. Nutrient Intake According to Dietary Cluster

Table 3 outlines the nutrient intake of the subjects according to their diet cluster. The macronutrient distribution was significantly different between the two dietary clusters, with a larger proportion of energy intake from carbohydrate in the hiSSB group and from fat and protein in the loSSB group. Macronutrient intakes for total energy and sugar were significantly higher in the hiSSB group and intakes for cholesterol were significantly higher in the loSSB group. Micronutrient intakes differed significantly between groups for vitamin C, zinc (Zn), chromium (Cr), and selenium (Se). With the exception of vitamin C, intakes for these nutrients were lower in the hiSSB group compared to the loSSB group.

Based on the Recommended Dietary Allowance (RDA), both clusters exceeded the Acceptable Macronutrient Distribution Range (AMDR) for fat [32] and the Dietary Guidelines for Americans’ (DGA) recommendations for both sodium and sugars [31]. Intakes for fiber, magnesium (Mg), Zn, vitamins C and E, Cr, and folic acid fell below RDA guidelines for both groups [32]. USDA My Plate recommendations fell below the recommended minimum serving amounts for all subjects for grains (76%), vegetables (33%), fruits (24%), and dairy (13%), while intakes exceeded minimum recommended servings of protein for both the hiSSB (132%) and loSSB (162%)clusters, [29].

### 3.5. KDQOL among MHD Patients According to Diet Clusters among MHD Patients

**Figure 1:**
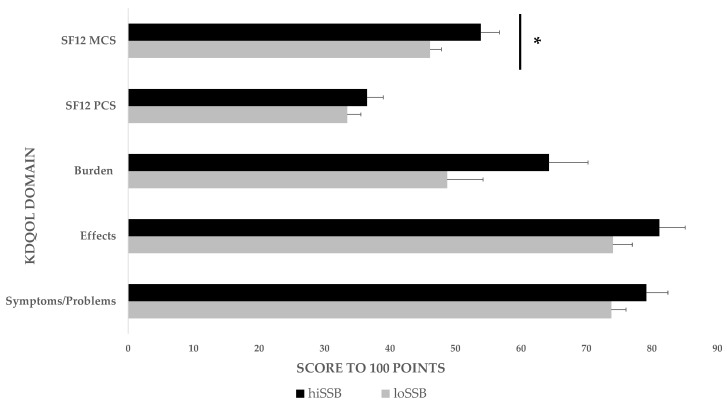
KDQOL scores according to diet cluster. SF 12 MCS: short form mental component summary; SF 12: short form physical component summary. Data is presented as means ± SEM. In comparison with patients consuming a loSSB pattern, those consuming a hiSSB pattern reported lower baseline KDQOL scores across all five domains and significantly lower baseline KDQOL scores for the S12 mental composite subscale. hiSSB, *n* = 20; loSSB, *n* = 28. Data were compared using a Mann–Whitney U test with a * p < 0.05 considered significant.

Those patients following a hiSSB diet pattern scored lower baseline values on all five KDQOL domains and significantly lower on the S12 mental composite domain (*p* < 0.026) (Table S1) compared to those following a loSSB pattern (Figure 1). Additionally, in a univariate analysis we compared KDQOL scores across each domain for additional variables (Table S2), and found that symptoms and effects scores were positively associated with age, the burden score was positively associated with vintage, and the PCS score was negatively associated with vintage.

## 4. Discussion

Our findings on this AA patient group on maintenance HD (AA-HD) are consistent with those of previous studies in non-CKD populations, which found that obesogenic dietary behaviors—such as a greater consumption of refined carbohydrates and fast foods, and low intakes of fruits and vegetables—were associated with lower self-reported QOL [33]. Alternatively, adherence to a healthy diet pattern, such as the Mediterranean diet, has been associated with better HRQOL, which may be partially explained by higher antioxidant content [34,35]. MHD patients are often instructed to avoid dairy products, fruits, vegetables, and whole grains in an effort to reduce phosphorus and potassium intake. At the same time, they are encouraged to consume protein rich foods, especially animal products with high biological value proteins. To meet energy requirements, juices, “clear” sugar sweetened carbonated beverages, and higher fat non-dairy products are often suggested as acceptable choices.

It is worth noting that only 3% of the entire study population consumed at least four servings of fruits and vegetables per day, and thus the majority failed to achieve the minimum four to five daily servings recommended by the USDA for women and men aged 31–50 [31]. Dietary intakes for fiber, Mg, Zn, Cr, folic acid, and vitamins C and E fell below RDA guidelines for both groups, which may be attributed to the low consumption of micronutrient rich foods. The percentage of calories from carbohydrates fell below the AMDR range for the loSSB group and was at the lower end of the range for the hiSSB group. Both groups failed to meet the USDA recommendations for grains, with refined carbohydrates exceeding DGA guideline upper limits.

Micronutrient intakes for Zn, Cr, and Se were significantly lower in the hiSSB. Previous studies have found that low intakes of Zn and Se have been associated with lower QOL indicators. Low Zn status has been associated with impaired QOL due to lower physical ability and fatigue in non-CKD patients [36,37]. Zn deficiency can contribute to disturbances in taste (dysgeusia) and smell, which may lead to poor nutritional intake, a commonly observed issue in CKD patients [38,39,40]. Non-CKD elderly persons with lower Se intakes and serum levels reported poorer self-perceived health and chewing ability [41]. Poor intakes of nutrients such as Zn and Se may have played a role in the lower QOL scores observed in the hiSSB group.

Inadequate micronutrient intake may have also contributed to the lower total HDL cholesterol, large HDL subfractions, and the atherogenic pattern B phenotype observed in the hiSSB group. Low Zn status has been associated with lipid peroxidation and inflammation in those with CKD [42]. Lower Cr levels have been associated with malnutrition in HD patients [43], and with inflammation, increased cardiovascular risk, and lower levels of HDL in non-CKD populations [44,45]. Lower concentrations of Se have been associated with higher rates of hospitalization and death among HD patients [46]. The hiSSB group tended to have higher levels of CRP, IL-18, and MCP-1, although the difference in these inflammatory markers between the loSSB and hiSSB groups was not significant. Given that low grade inflammation may play a role as both a cause and consequence of low HDL-C levels [47,48], and higher HDL-C levels may act as a buffer against low-grade inflammation [49,50,51], the lower levels of HDL-C observed in the hiSSB group may have been a consequence of proinflammatory factors modulated by a high sugar dietary pattern. 

Although the burden of following a restrictive diet has the potential to impair QOL [52], it has been found that HD patients who control their diet benefit from reduced symptom burden and enhanced general health and wellbeing [53]. In non-dialysis populations, following healthy dietary patterns has also been associated with improved QOL [54,55]. In the *African American Study of Kidney Disease and Hypertension*, both lower mental and physical health scores were associated with increased risk of cardiovascular events among blacks with hypertensive CKD. Researchers postulated that the lower QOL scores may have been attributed to several factors including poor self-care, resulting in poor compliance to treatment regimens such as prescribed medical nutrition therapy [56,57]. Additionally, Feroze et al. found that not only were lower mental health scores the most powerful predictors of mortality—with each 10-unit lower score associated with an approximately 12% higher death risk—but that these low scores were better mortality predictors among AA-HD patients compared to whites. Potential contributors to reduced QOL for MHD patients include surrogates of protein energy wasting, obesity, and higher levels of proinflammatory cytokines [58,59].

Unfortunately, there has been little improvement in HRQOL among HD patients over the past decade [60,61]. HD patients who are consuming an energy dense diet which relies heavily on hiSSB and fast foods may be lacking in several micronutrients [62]. Most renal specific vitamins, including the brands prescribed to the patients in this study, provide the water-soluble B vitamins and vitamin C lost through dialysis. Only a few renal specific vitamins provide additional micronutrients such as vitamin E, Se, and Zn. These patients may benefit from targeted nutritional intervention or supplementation.

Our study has important strengths, such as the inclusion of patients from multiple HD centers and the use of rigorous analytical and laboratory methods. However, this study has some limitations. First, diet data was not collected for dialysis days, and may not represent usual intake. Second, the small sample size and homogenous population can impact the ability to robustly detect differences between groups. Third, the KDQOL is not validated to specifically measure the impact of diet on health; however, to our knowledge, there is no specific instrument that measures dietary contribution to HRQOL for CKD patients. Fourth, causality cannot be determined due to the cross-sectional nature of this study. Therefore, it is unclear whether dietary patterns may have influenced QOL or if self-perceived QOL affected dietary choices. Finally, micronutrient values may have been underestimated, since not all commercial products in ESHA food processor software have complete information.

## 5. Conclusions

Lower KDQOL scores among MHD patients were associated with a dietary pattern characterized by high intakes of sugar sweetened beverages and reduced intakes of protein foods and vegetables. The clinical impact of excessive refined sugar intake was also associated with significantly lower intakes of several micronutrients, including Zn, Cr, and Se, potentially contributing to the lower KDQOL scores observed as well as lower levels of HDL cholesterol and large HDL subfractions. Further studies examining the role of dietary micronutrients as antioxidants with lipoprotein modification is warranted.

## 6. Practical Implication

Renal dietary guidelines generally focus on specific nutrients and foods to avoid in an effort to control serum phosphorus, potassium, and fluid, while at the same time encouraging adequate protein intake to avoid negative nitrogen balance. Less attention is given to diet-derived micronutrient intake. We found that a dietary pattern high in refined sugars can have a negative impact on several health outcomes, including QOL and lipid profiles in an African American HD cohort. Future studies examining diet patterns associated with improved outcomes in the HD population are needed.

## Figures and Tables

**Table 1:** Percentage of energy contribution of food groups.

	Pattern 1 (hiSSB)	Pattern 2 (loSSB)	
Food Groups	Mean (SD) %TE Food		*p*
Sugar sweetened beverages	27.97 ± 9.27	9.45 ± 5.65	<0.001
Unprocessed red meat	0.95 ± 1.49	2.17 ± 3.49	0.022
Poultry	4.67 ± 5.99	2.51 ± 2.47	0.024
Fish and shellfish	0.49 ± 0.93	1.42 ± 2.87	0.028
Puddings, ice cream, cheesecake	0.24 ± 0.78	0.76 ± 1.70	0.049
Processed and cured meats (bacon, sausage, hot dogs)	2.67 ± 2.47	3.62 ± 3.39	0.106
Dairy, low-fat and 2%	1.75 ± 4.61	0.59 ± 2.30	0.122
Egg and egg dishes	2.43 ± 2.60	3.29 ± 3.08	0.132
Vegetables, canned, fresh and frozen	3.25 ± 4.20	4.45 ± 4.11	0.151
Fast foods, frozen and convenience entrees	4.46 ± 6.67	2.92 ± 4.87	0.194
Pizza, pasta and lasagna	2.77 ± 4.95	4.25 ± 6.58	0.200
Butter, margarine, animal fats	0.25 ± 0.41	0.41± 0.95	0.256
Potatoes, mashed and salad	0.87 ± 2.16	1.39 ± 2.55	0.270
Beans and legumes	0.43 ± 1.25	0.75 ± 1.92	0.315
Potatoes, fried and hash browns	1.39 ± 2.26	0.96 ± 2.18	0.330
Fruit, canned, fresh and dried	2.02 ± 4.61	1.28 ± 2.53	0.335
Oils (vegetable, olive, canola)	0.03 ± 0.09	0.05 ± 0.20	0.398
Crackers, chips and popcorn	2.12 ± 4.80	1.60 ± 2.57	0.509
Candy	0.47 ± 1.11	0.34 ± 1.01	0.542
Sauce and condiments, savory	0.83 ± 1.50	0.69 ± 0.92	0.597
Nuts and seeds and nut butters	0.24 ± 0.84	0.17 ± 0.86	0.665
Dairy, full-fat and creamer	1.00 ± 3.00	0.75 ± 3.18	0.691
Cakes, cookies, pie, donuts, and rich dough	1.71 ± 2.34	1.78 ± 2.70	0.885
Grains	6.38 ± 8.63	6.62 ± 8.26	0.888
Pork	0.71 ± 2.22	0.76 ± 1.65	0.889
Sauces and condiments, sweet	0.40 ± 0.62	0.39 ± 0.68	0.945

Values are mean ± SD; *n* = 47 for hiSSB and 54 for the loSSB group. %TE food: the percentage total energy contribution from food.

**Table 2:** Patient characteristics at baseline according to diet cluster.

	All (*n* = 100 ^†^)	hiSSB (*n* = 47)	loSSB (*n* = 53)	*p* Value between Groups
Age, year	60 (53–68)	59 ± 12	60 ± 14	0.662
Ethnicity				
African American, *n* (%)	100 (100)	47 (47)	54 (53)	---
Males, *n* (%)	59 (59)	29 (29)	30 (30)	0.605
BMI, kg/m^2^	27.4 (23.3–31.4)	29.7 ± 6.7	26.9 ± 6.0	0.029
BMI Category, *n*(%)				
Underweight (BMI < 18.5)	3 (3)	1 (1)	2(2)	0.028
Normal Weight (18.5–24.9)	31 (31)	8 (8)	23 (23)	
Overweight (BMI 25–29.9)	31 (31)	19 (19)	12 (12)	
Obese (BMI > 30)	35 (35)	19 (19)	16 (16)	
Vintage (months)	45 (19–88)	62 ± 57	66 ± 68	0.770
Cause of Kidney Failure				
Diabetes Mellitus, *n* (%)	47 (47)	24 (24)	23 (23)	0.663
Hypertension, *n* (%)	37 (37)	18 (18)	19 (19)	
Glomerulonephritis, *n* (%)	5 (5)	2 (2)	3 (3)	
SLE, *n* (%)	2 (2)	0 (0)	2 (2)	
HIV-Nephropathy, *n* (%)	2 (2)	0 (0)	2 (2)	
Others, *n* (%)	5 (5)	2 (2)	3 (3)	
Unknown, *n* (%)	2 (2)	1 (1)	1 (1)	
Insulin Use, *n* (%)	30 (30)	20 (20)	10 (10)	0.010
Oral Hypoglycemic Agent Use, *n* (%)	8 (8)	2 (2)	6 (6)	0.194
Tobacco Use, *n* (%)	29 (29)	14 (14)	15 (15)	0.870
Vascular Access				
Arteriovenous fistula, *n* (%)	59 (59)	28 (28)	31 (31)	0.993
Arteriovenous graft, *n* (%)	26 (26)	12 (12)	15 (14)	
Catheter, *n* (%)	15 (15)	7 (7)	8 (8)	
Blood Pressure, mmHg (post-sitting)				
^1^ Systolic	138 (118–155)	141 ± 24	139 ± 21	0.620
^1^ Diastolic	77 (70–83)	78 ± 10	80 ± 22	0.483
^2^ Kt/V	1.5 (1.4–1.6)	1.5 ± 0.2	1.6 ± 0.3	0.084
Antidepressant use, %	17 (17)	12 (12)	5 (5)	0.032
Renal vitamin * use, %	56 (56)	24 (24)	32 (32)	0.349
Serum Potassium (mEq/L)	4.6 (4.1–5.1)	4.7 ± 0.6	4.6 ± 0.6	0.632
Serum Phosphorus (mg/dL)	5.0 (4.3–5.8)	5.2 ± 1.5	5.0 ± 1.0	0.285
^3^ Serum Albumin (g/dL)	3.8 (3.6–4.0)	3.8 ± 0.4	3.8 ± 0.3	0.948
^4^ CRP (mg/L)	6.1 (3.1–8.4)	6.2 ± 4.8	6.0 ± 3.2	0.745
^4^ IL-6 (pg/mL)	1.7 (0.01–5.3)	10.9 ± 36.7	13.2 ± 54.4	0.799
^5^ IL-18 (pg/mL)	238 (172–320)	276 ± 146	259 ± 163	0.594
^4^ MCP-1 (pg/mL)	113 (89–160)	155 ± 127	134 ± 97	0.373
Total Cholesterol (mg/dL)	148 (113–188)	147 ± 41	154 ± 47	0.471
Triglycerides (mg/dL)	81 (53–125)	106 ± 52	87 ± 51	0.069
LDL-C (mg/dL)	76 (46–104)	80 ± 34	80 ± 45	0.987
HDL-C (mg/dL)	47 (39–62)	46 ± 16	56 ± 21	0.007
Large HDL (mg/dL)	17.0 (11.0–31.8)	17.9 ± 12.7	27.2 ± 17.8	0.003
Intermediate HDL (mg/dL)	23.0 (18.3–26.0)	21.7 ± 5.4	23.8 ± 6.1	0.081
Small HDL (mg/dL)	6.0 (4.0–8.0)	6.7 ± 3.1	5.5 ± 3.4	0.076
^6^ Large LDL(mg/dL)	20.5 (14.0–28.0)	20.4 ± 9.2	23.2 ± 10.1	0.154
^6^ Intermediate LDL (mg/dL)	11.0 (7.0–16.0)	13.7 ± 7.8	11.8 ± 8.4	0.251
^6^ Small LDL (mg/dL)	2.0 (0.0–5.3)	4.9 ± 6.1	3.1 ± 4.8	0.103
^6^ Mean LDL Size (Å)	270.0 (266.0–273.0)	268.0 ± 4.6	270.3 ± 4.1	0.009
LDL Pattern A, *n* (%)	62 (61.4)	22 (21.8)	40 (39.6)	0.050
LDL Pattern B, *n* (%)	16 (15.8)	10 (9.9)	6 (5.9)	0.050

^†^ Excludes 1 hypertriglyceridemic subject. Values are expressed as median (Q1–Q3), means ± SDs, or percentages. Statistics: 2-sample *t* test, or Pearson chi-square test. BMI: Body Mass Index;; SLE: Systemic lupus erythematosus; Kt/V is a number used to quantify hemodialysis (K, dialyzer clearance of urea; t, dialysis time; V, volume of distribution of urea, approximately equal to patient’s total body water); * Renal vitamins included Nephrocaps, Renal Caps, and Nephrovite brands; CRP: C-reactive protein; IL-6: Interleukin 6; IL-18: Interleukin 18; MCP-1: Monocyte Chemoattractant Protein-1; HDL: high density lipoprotein cholesterol; LDL: low density lipoprotein cholesterol; Å: angstrom; ^1^ BP hiSSB (*n* = 44), loSSB (*n* = 43) ^2^ Kt/V hi SSB (*n* = 43), loSSB (*n* = 45); ^3^ Albumin hiSSB (*n* = 46), loSSB (*n* = 50); ^4^ CRP, ^4^ IL-6, ^4^ MCP-1 hiSSB (*n* = 46), loSSB (*n* = 52); ^5^ IL-18 hiSSB (*n* = 46), loSSB (*n* = 49); ^6^ large, ^6^ intermediate, and ^6^ small LDL, ^6^ mean LDL size and ^6^ LDL pattern hiSSB (*n* = 46), loSSB (*n* = 52).

**Table 3:** Mean daily nutrient intake according to diet cluster.

Nutrient	All (*n* = 100 ^†^)	hiSSB (*n* = 47)	loSSB (*n* = 53)	*P* Value between Groups	Nutrient Recommendations
Energy, kcals	2027 ± 414	2123 ± 432	1941 ± 381	0.029	Per renal Rx
Protein, g	86 ± 26	83 ± 21	88 ± 29	0.311	Per renal Rx
% kcals from protein	17 ± 4	16 ± 4	18 ± 4	0.008	10–35 *
Fat, g	90 ± 24	89 ± 25	91 ± 24	0.713	
% kcals from fat	40 ± 7	38 ± 6	42 ± 7	0.001	20–35 *
CHO, g	218 ± 65	249 ± 68	191 ± 50	<0.001	
% kcals from CHO	43 ± 8	47 ± 7	40 ± 7	<0.001	45–65 *
Fiber, g	12 ± 4	13.2 ± 3.9	11.8 ± 4.5	0.095	30 *
Sugars, g	87 ± 45	115 ± 47	63 ± 23	<0.001	<50 **
Phosphorus, mg	833 ± 303	797 ± 260	864 ± 335	0.263	Per renal Rx
Iron, g	11.5 ± 4.5	11.3 ± 4.1	11.6 ± 4.9	0.800	Per renal Rx
Magnesium, mg	149 ± 64	150 ± 65	147 ± 64	0.852	420 *
Sodium, mg	2996 ± 961	3036 ± 1013	2960 ± 921	0.695	<2300 **
Potassium, mg	1500 ± 585	1487 ± 636	1512 ± 542	0.834	Per renal Rx
Zinc, mg	8.5 ± 4.1	7.4 ± 2.9	9.4 ± 4.8	0.017	11 *
Vitamin C, mg	58 ± 46	69 ± 55	48 ± 33	0.023	90 *
Vitamin E, mg	4.3 ± 3.1	4.4 ± 3.4	4.1 ± 2.9	0.628	15 *
Chromium (µg)	4.4 ± 6.9	2.9 ± 4.5	5.7 ± 8.2	0.031	30 *
Selenium (µg)	88.2 ± 37.8	79.6 ± 35.3	95.8 ± 38.7	0.023	55 *
Folic acid, mg	225 ± 108	230 ± 112	221 ± 105	0.707	400 *
Cholesterol, mg	412 ± 177	368 ± 153	452 ± 188	0.016	Per renal Rx or (<200) [28]
USDA My plate recommendations (%)					
Grain	76 ± 33	75 ± 37	77 ± 30	0.749	Based on age and gender [29]
Vegetable	33 ± 27	30 ± 25	35 ± 28	0.369	
Fruit	24 ± 33	32 ± 41	16 ± 21	0.023	
Dairy	13 ± 14	15 ± 14	12 ± 15	0.306	
Protein	148 ± 64	132 ± 59	162 ± 65	0.019	

^†^ Excludes 1 hypertriglyceridemic subject. Values are means ± SDs, or percentages. Statistics: 2-sample *t* test, or Pearson chi-square test. * DRI, Dietary Reference Intakes for males ages 51–70 [30]; CHO, carbohydrate; Rx, prescription; ** 2015–2020 Dietary Guidelines for Americans recommends <10 percent of calories per day from added sugars or 50 grams for a 2000 kcal [31].

## Supplementary Materials

The following are available online at https://www.mdpi.com/2072-6643/12/3/797/s1, Table S1: KDQOL scores according to diet cluster. Table S2: Comparison of KDQOL score by selected variables.
