# Correction: Tallman, D.A., et al. Dietary Patterns and Health Outcomes among African American Maintenance Hemodialysis Patients. *Nutrients* 2020, *12*(3), 797

**DOI:** 10.3390/nu12113430

**Published:** 2020-11-09

**Authors:** Dina A. Tallman, Eno Latifi, Deepinder Kaur, Ayesha Sulaheen, T. Alp Ikizler, Karuthan Chinna, Zulfitri Azuan Mat Daud, Tilakavati Karupaiah, Pramod Khosla

**Affiliations:** 1 Department of Nutrition and Food Science, Wayne State University, Detroit, MI 48202, USA; fq8257@wayne.edu (D.A.T.); Eno.Latifi@wayne.edu (E.L.); kdeepinder@wayne.edu (D.K.); 2 Dietetics Program, Faculty of Health Sciences, Universiti Kebangsaan Malaysia, Kuala Lumpur 43600, Malaysia; aishaltaf@ymail.com; 3 Division of Nephrology and Hypertension, Vanderbilt University Medical Center, Nashville, TN 37232, USA; alp.ikizler@Vanderbilt.Edu; 4 School of Medicine, Faculty of Health Sciences, Taylors University, Subang Jaya 47500, Malaysia; karuthan@gmail.com (K.C.); tilly_karu@yahoo.co.uk (T.K.); 5 Department of Nutrition and Dietetics, Faculty of Medicine and Health Sciences, Universiti Putra Malaysia, Serdang 43400, Malaysia; zulfitri@upm.edu.my

The authors wish to make the following correction to this paper [1].


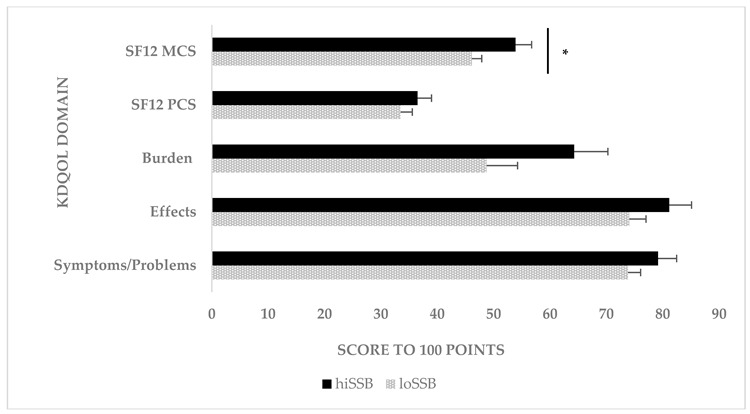


The chart element colors were inadvertently mislabeled. Please replace the original figure above with the corrected figure below. 


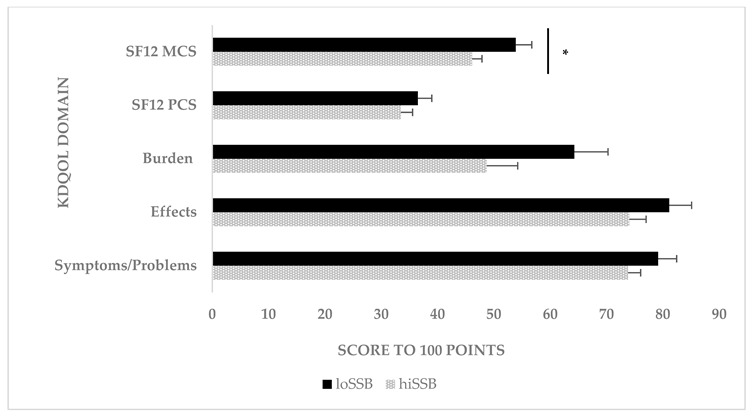


These changes have no material impact on the conclusions of our paper. We apologize for any inconvenience to the readers of *Nutrients*. The published version will be updated on the article webpage, with a reference to this correction notice.
