# Supplementary material for: Dietary Patterns and Health Outcomes among African American Maintenance Hemodialysis Patients
Source: Nutrients. 2020 Mar 18;12(3):797. doi: 10.3390/nu12030797 (PMC7146457; doi:10.3390/nu12030797)
Supplement: Supplementary file 1 [file nutrients-12-00797-s001.pdf]

Table S1. KDQOL scores according to diet cluster.

	hiSSB (n=20)	loSSB (n = 28)	*P* value between groups
**Average Symptom KDQOL**	77 (68 - 82)	81 (73 -88)	0.228
**Average Effects KDQOL**	73 (63 - 89)	81 (70 -94)	0.176
**Average Burden KDQOL**	41 (25 - 72)	69 (31 -88)	0.058
**Average S12 PCS KDQOL**	33 (27 -42)	38 (29 -44)	0.363
**Average SF MCS KDQOL**	47 (39 - 58)	57 (48 -61)	0.026

Data is presented as Median (Q1-Q3); Mann-Whitney U test

Table S2: Comparison of KDQOL score by selected variables

**Variable**	Symptoms	Effects	Burden	PCS	MCS
**Diet Cluster**					
**hiSSB**	73.8±13.1	74.1±17.7	48.8±26.7	33.5±11.1	46.1±12.7[a]
**loSSB**	79.2±10.7	81.1±15.6	64.8±29.0	36.5±11.0	53.0± 9.2[a]
**Gender**					
**Male**	77.4±11.6	78.2±16.8	56.3±28.6	35.4±11.7	50.7±12.5
**Female**	76.5±12.6	78.1±17.1	59.8±29.7	35.0±10.3	50.6±10.0
**Tobacco use**					
**Yes**	74.8±14.1	77.9±21.5	56.3±33.2	35.0±10.7	50.6±12.6
**No**	77.9±10.9	78.3±14.4	58.5±27.1	35.3±11.3	50.7±11.0
**Antidepressant use**					
**Yes**	66.1±17.6	66.3±24.9	42.5±29.1	28.0±9.2	43.6±10.9
**No**	78.2±10.7	79.6±15.3	59.6±28.6	36.1±11.0	51.5±11.3
**Age [β(se)]**	0.36(0.15)*	0.52(0.19)*	0.32(0.36)	0.03(0.13)	0.27(0.14)
**Vintage [β(se)]**	-0.03(0.03)	0.06(0.03)	0.13(0.06)*	-0.06(0.02)*	0.01(0.02)
**BMI [β(se)]**	-0.03(0.35)	-0.20(0.45)	0.56(0.80)	-0.35(0.28)	-0.46(0.30)

[a] significant difference at 0.05. *significant association at 0.05. Values are mean ± SD. BMI: Body Mass Index.
